# Peptides and proteins with antimicrobial activity

**DOI:** 10.4103/0253-7613.40481

**Published:** 2008

**Authors:** Henrique Douglas Melo Coutinho, Katiuscia Menezes Lôbo, Denise Aline Casimiro Bezerra, Inalzuir Lôbo

**Affiliations:** Departamento De Ciências Físicas E Biológicas - DCFB, Centro De Ciências Biológicas E Da Saúde - CCBS, Universidade Regional Do Cariri - URCA, Brazil; 1 Departamento De Enfermagem - De, Centro De Ciências Biológicas E Da Saúde - CCBS, Universidade Regional Do Cariri - URCA, Brazil

**Keywords:** Antimicrobial activity, bacteriocin, defensin, peptides

## Abstract

The increase of microbial resistance to antibiotics has led to a continuing search for newer and more effective drugs. Antimicrobial peptides are generally found in animals, plants, and microorganisms and are of great interest to medicine, pharmacology, and the food industry. These peptides are capable of inhibiting pathogenic microorganisms. They can attack parasites, while causing little or no harm to the host cells. The defensins are peptides found in granules in the polymorphonuclear neutrophils (PMNs) and are responsible for the defense of the organism. Several animal defensins, like dermaseptin, antileukoprotease, protegrin, and others, have had their activities and efficacy tested and been shown to be effective against bacteria, fungi, and protists; there are also specific defensins from invertebrates, e.g., drosomycin and heliomicin; from plants, e.g., the types A and B; and the bacteriocins, e.g., acrocin, marcescin, etc. The aim of the present work was to compile a comprehensive bibliographic review of the diverse potentially antimicrobial peptides in an effort to systematize the current knowledge on these substances as a contribution for further researches. The currently available bibliography does not give a holistic approach on this subject. The present work intends to show that the mechanism of defense represented by defensins is promising from the perspective of its application in the treatment of infectious diseases in human, animals and plants.

A substance to be deemed a useful chemotherapeutic agent must have toxic selectivity for the parasite; this means that, at an effective concentration in the tissues, the substance must have low toxicity to host cells and high toxicity to the infective agent; it also should not alter the natural defense mechanisms of the host, such as phagocytosis and synthesis of antibodies.[1] One of the aims of the present work is to evaluate natural substances (antimicrobial peptides) that have chemotherapeutic activity.

Antimicrobial peptides present new possibilities for combating infectious diseases. They inhibit the growth of pathogenic microorganisms, without affecting the host or the animals and plants that produce them, and have a broad spectrum of antimicrobial activity. It is well known that bacteria, induced by stress, produce bacteriocins that may cure infectious diseases.[2] Peptides found in some animals may also affect microorganisms; for example, protegrin, a porcine defensin, acts against *Escherichia coli, Listeria monocytogenes,* and *Candida albicans.*[3]

These peptides may be found in all living organisms; they can be modified in the laboratory or they can be obtained from organisms that produce them for their own defense. For example, antimicrobial activity has been assigned to the secretion from the salivary glands of termites and has been shown to have antifungal action.[4]

We have chosen this subject hoping to put together the still limited knowledge on several antimicrobial peptides of many animals and plants and their respective actions. Peptides have shown to be as effective as, or even more effective than, drugs synthesized in the laboratory to which many microorganisms have developed resistance. For example, the malarial protozoan has developed resistance to many insecticides and the peptide dermaseptin has been demonstrated to have antimalarial activity.[5]

There is increasing concern about the resistance of microorganisms to various drugs and the perspective of continuous use of antibiotics is not yet well defined. Therefore, many measures to solve this problem need to be adopted, e.g., the controlled use of antibiotics, expansion of research for the better understanding of the resistance mechanisms, and continuing attempts to develop new synthetic and natural drugs.[6] Peptides, we believe, constitute a novel potential therapeutic agent against diseases caused by pathogenic organisms.

It is aimed in the present work to put together a bibliographical review on the antimicrobial potential of peptides from several living organisms. We have carried out a literature survey to identify the potential uses and origins of these substances that are found in neutrophils, salivary glands, bacteria, fungal cells, etc. and have the ability to inhibit several pathogens.

## Materials and Methods

We searched the international bibliographic data banks SciELO, HighWire, PubMed, BIREME, and Lilacs, using the key words ‘peptides,’ ‘bacteriocins,’ ‘defensins,’ and ‘antimicrobial activity.’ The bibliographical search covered the period from the 90s up to December 2006 and also included the more recent articles of 2007.

## The use of Substances Obtained from Microorganisms

It is well known that some microorganisms inhibit or destroy others. These microorganisms are now being studied with respect to their capacity to produce substances that attack specific cells. Despite many studies having been performed on the antimicrobial activities of animals and plants, few have been widely published.[7] This is a broad field of investigation; peptides and proteins have been widely used in the pharmaceutical and food industries and in medical therapy due to their huge biological activity. Bacteria generally have the genetic ability to acquire resistance to drugs used as therapeutic agents and to transmit this to other bacteria.

Substances with antimicrobial activity are comprised of peptides or proteins that affect the pathogenic microorganism. Some peptides have intense biological activity; they are chains of amino acids linked by a peptide bond (amide group). Proteins are made up of a set of 20 amino acids linked covalently together in a linear sequence; they have many different properties and activities, viz, enzymes, transport proteins, and nutritional proteins. Among these latter are organism-feeding substances, like the egg-white protein and monellin (isolated from the fruit of *Dioscoreophylum cumnisii,*[8] which tastes sweet and is used as an atoxic and low calorie sweetener). Among the many proteins are the contractile proteins that are involved in movement, structural proteins, defense proteins, regulators, and repressor proteins.

## Antimicrobial Activity of Peptides from Vertebrates

The protozoan *Plasmodium*, which causes malaria, is becoming more resistant nowadays due to the continuous use of insecticides for exterminating its vector, the anopheles mosquito, the female individuals of which are responsible for infecting millions of people in the world. It has been reported[5] that 1.5-2 million children die every year in Africa from malaria.

Studies show that the hemolytic antimicrobial peptide dermaseptin S4 inhibits the growth of the malarial parasite. Peptides that are potentially active against a broad spectrum of microorganisms are generally nontoxic to cells of mammals. Dermaseptin belongs to a large family of proteins produced in the skin of frogs of the genus *Phyllomedusinae*. Such proteins have cytolytic activity *in vitro* (against bacteria, protozoans, filamentous fungi, and yeasts); their derivatives (S4, K4-S4, K_4_K_20,_ -S4, and D_4_D_20_ -S4) are also active and are able to disrupt the plasmatic membrane of the parasite and to damage the parasite when it is outside the red blood cell. Among the derivatives, K_4_-S4 is the most effective, having a ten-fold greater antimalarial activity, which means it can be used as a component of a new drug against the *Plasmodium.*[5]

The human respiratory tract represents a way into the body for airborne microorganisms. In defense against this, leukocytes produce antileukoprotease (ALP) and the liver produces the α-1 proteinase, which reach the lungs by passive diffusion. ALP and α-1 proteinase are inhibitors of serine proteinase that is present in various external secretions. The former has antibacterial activity against *Escherichia coli* and *Staphylococcus aureus*, being characterized in the NH_2_-terminal domain. The COOH-terminal domain present in the structure inhibits the elastase, that causes tissue degradation stimulated during inflammation by neutrophils.[9] Antibacterial cationic polypeptide, eNAP-2, produced from equines by neutrophils, was identified and shown to have the same characteristics as ALP. An inhibitory proteinase, designated as aprotinin, a protein that is present in the lungs and other organs of bovines and has activity against Gram-positive and Gram-negative bacteria has also been reported.[9]

In human lungs, cells from the alveolar epithelium, non-ciliated bronchiolar cells, and some epithelial cells synthesize structures homologous to Ca^++^-dependent lectins, which are designated as collectins (SP-A and SP-D, surfactant proteins A and D) and which play a role in the defense of the organism. SP-A was tested in transgenic mice and caused a decrease of group B streptococci (GBS) strains, *S. aureus,* and *Pseudomonas aeruginosa* after intratracheal inoculation; SP-D is known for its action on LPS (lipopolysaccharide) of Gram-negative bacteria.[10]

Each cell is self-protected against specific pathogens. In mammals, the peroxidase system shows activity against Gram-negative and Gram-positive bacteria, fungi, and viruses. This system is important for inhibiting tooth decay, controlling microorganisms in the milk of animals during the period of lactation, and for mediating and killing pathogenic cells.[11] Three peroxidases have been detected: LPO (lactoperoxidase), found in various external and internal secretions, e.g., saliva, milk, tears, and in the fluids lining the airways; MPO (mieloperoxidase), found in neutrophils, monocytes, eosinophil peroxidase and thyroidal peroxidase; and APO (peroxidase in the airways), found in pulmonary fluids[11] [Table 1].

**Table 1:** Antimicrobial activity of peptides and defensins from vertebrates

*Name*	*Origin*	*Action*	*Similarity*	*Reference*
Dermaseptin	Frogs of genus	Bacteria, Protozoans, Fungi, Yeasts		5
	Phyllomedusidae			
Antileukoprotease (ALP)	Human leukocyte	Gram-positive and Gram-negative bacteria	eNAP α-1 proteinase	12
α-1 proteinase	Human liver	Gram-positive and Gram-negative bacteria	ALP eNAP	13
Aprotinin	Bovine lung	Gram-positive and Gram-negative bacteria		9
Collectins	Human lung	Streptococcus Gram-negative bacteria		10
Lactoperoxidase (LPO)	Peroxidase system in mammals	Gram-positive and Gram-negative bacteria		11
Mieloperoxidase (MPO) Peroxidase in airways (APO)		Fungi Virus		
**Defensins**				
HNPs	Neutrophils (Human and rabbits)	Bactericide Fungi Virus	Protegrin (PGs)	14
HD (Enteric defensins)	Intestinal tract	Bactericide		15
hβD	Epithelium	Bactericide		15
tPMP-1	Platelets (Megakaryocyte)	*S aureus*	HNP-1	16,17
RTD and RTD-1, (θ defensins from Rhesus)	*Rhesus macaque*	*S aureus, Candida albicans, Cryptococcus neoformans, E coli* (RTD- 2)	Protegrin (PGs), Tacplesin	18
(PGs) Protegrin	Porcine leukocytes	*E coli, L monocytogenes, C albicans,* (*in vitro*)	RTD-1	15

## Antimicrobial Activity of Bacterial Peptides

*Lactobacillus* is widely used for fermenting natural (especially dairy) products and also in pharmaceuticals. The lactobacilli have been studied for activity in the prevention and treatment of gastrointestinal diseases, the exogenous *Lactobacillus* is believed to exert a ‘barrier effect’ against pathogens. The *L. casei* GG can be used in diarrhea in human and *L. gasseri* ADH has great metabolic effects on decreasing the enzymatic fecal activity. Another *Lactobacillus* with antibacterial activity is *L. reuteri* MM53, isolated from human milk, that is able to survive in the gastrointestinal tract.[19]

It is noteworthy that all these acid lactic bacteria have in common the property to inhibit pathogenic microorganisms. Bernet-Camard and collaborators reported antibacterial activity in intestinal cells *in vitro*. Substances secreted by *Lactobacillus acidophilus*-LA1 inhibit enteric bacteria activity. The suspension from LA1 culture (LA1-SCS) produces antibacterial components active against *Salmonella typhimurium* that infects cultures of human intestinal cells designated as Caco-2 (the adenocarcinoma cell of the human colon).[19] It was also observed that LA1-SCS acts *in vitro* against pathogenic Gram-positive and Gram-negative bacteria, namely *S. aureus, Listeria monocytogenes, S. typhimurium, Shigella flexneri, Klebsiella pneumoniae, P. aeruginosa,* and *Enterobacter cloacae*. LA1, when administered orally to humans through fermented milk, enhances phagocytic activity in the blood.[19]

## Defensins from Vertebrates

In mammals, the defensins constitute one of the largest families of antimicrobial peptides.[18] The defensins identified in humans are: 4α defensins of neutrophils (HNP-1 to 4); 2α enteric defensins (HD-5 and HD-6); and 4β epithelial defensins (hβD-1 to 4). The defensins hβ2, 3 and 4 are induced by inflammatory cytocins or bacterial infection and hβ1 is expressed in the epithelium without induction.[20]

Defensins are small bacterial cationic peptides which are quite abundant in the granules of polymorphonuclear neutrophils (PMNs). Studies have shown that human PMNs have four defensins (HNP-1 to HNP-4). The HNPs 1, 2, and 3 are arginine-rich (amino acid) and have six residues of cystines, making up 5-7% of the protein in PMNs of humans.[15]

The total HNPs, HNP-1, and HNP-2, are bactericides with similar effects, whereas HNP-3 has lower activity. Takemura and collaborators[20] compared the effects of human defensins and estimated the time for bacterial death. They reported that human defensins are more active against *E. coli* ATCC 29648 and ML-35 than against *P. aeruginosa* PAO 579. This natural antibiotic has similar action in animals. It was observed in other experiments that human defensins are active *in vitro* against Gram-positive and Gram-negative bacteria, fungi, enveloped viruses, and other pathogens.[15]

Miyakawa and collaborators[15] reported that *Mycobacterium tuberculosis* H37Ra does not grow when cultivated in the presence of HNP-1, synthetic rabbit neutrophil peptide 1 (HNPs), or porcine protegrin 1 (PGs); thus, these peptides were shown to have an antibacterial effect. The authors also used scanning electron microscopy to show that these defensins caused lesions on the surface of H37Ra.

The human neutrophil defensins NHP-1, 2, and 3 have activity against *M. tuberculosis*, *Mycobacterium avium,* and *Mycobacterium intracelulari*. Biological activity against several pathogenic fungi was observed with HNP-1.[15]

Another cationic peptide is the thrombin-induced platelet protein-1 (tPMP-1), which has a broad spectrum of action against human pathogens, including *S. aureus*. This peptide, like HNP-1, attacks the bacterial membrane.[16]

The macrocyclic amino acid antimicrobial peptide termed rhesus θ-defensin 1 (RTD-1) and their derivatives (RTD1a and RTD1b) were the first to be discovered. The structures of RTD-2 and RTD-3 are the predicted homodimeric splicing products of RTD1b and RTD1a, respectively, as they were observed *in vivo.*[18] RTD-1, RTD-2, and RTD-3 have similar antibacterial activity against *S. aureus, C. albicans,* and *Cryptococcus neoformans* and RTD-2 are active against *E. coli*. These antimicrobial peptides are essential components of the innate immune system and have a significant role at the epithelial defense barrier and as part of the antibacterial arsenal of neutrophils and macrophages.[18]

There is evidence that RTD-1 has a defensive role in animals and is ten times more abundant than the peptides mentioned above, being similar to protegrin (the porcine defensin) and to tacplesin (the crab defensin). RTD-1 to RTD-3 have a broad spectrum of microbicidal action.[18]

Protegrin (PG) is a cystine-rich defensin of a new family of small AMPs (antibacterial endogenous peptides) obtained from porcine leukocytes. PG1, PG2, and PG3 have antibacterial activity *in vitro* against *E. coli*, *L. monocytogenes,* and *C. albicans.*[15]

## Defensins from Invertebrates

A crop pest that has been spreading over large areas of cotton plantations is the lepidopterous larva that attacks roots, leaves, flowers, and the cotton bolls. Some species, e.g., *Pestinophora gassypiella, Heliothis armigera, Heliothis virescens,* and Earias spp. have caused big losses to industry. More than 150 antimicrobial peptides from several insect species are currently known.[21]

Lepidoptera exhibit antimicrobial activity as a reaction to septic injury. Lamberty and collaborators[21] showed that the larva of *H. virescens* produce a cysteine-rich molecule with specific antifungal activity. These antimicrobial molecules are produced in the fat body (the functional equivalent of the mammalian liver) of lepidopterans. These defensins from invertebrates are grouped into four families: (i) cecropin, an antibacterial and antifungal cationic peptide isolated from *Hyalophora cecropia* and *Aedes aegypti*; (ii) cysteine-rich peptides with an amide terminal group (antibacterial) (defensins from insects are included in this group); this family of defensins is generally active against Gram-positive and Gram-negative bacteria. The drosomycin from *Drosophila melanogaster* and thanatin from *Podisus maculiventris* have antifungal and antibacterial activity are also included in this group; (iii) proline-rich peptides have been isolated from Hymenoptera, Diptera, Lepidoptera, and Hemiptera, which are active against Gram-negative bacteria and fungi; (iv) glycine-rich peptides/polypeptides designated as attacins and diptericins from lepidopterans and dipterans, and sarcotoxins II.

Gloverin and moricin were isolated from the lepidoterans *Hyalophora cecropia* and *Bombyx mori*, respectively; they are both antimicrobial defensins that are not included in any of the four groups mentioned previously. From *H. virescens* a new antifungal peptide was isolated, whose structure is similar to defensins from insects of the family II, e.g., drosomycin. This new defensin, the heliomicin, has a potent antifungal effect even in small concentrations.[21]

In 2001, Lamberty and collaborators[4] performed an analyses on two antimicrobial peptides isolated from the termite *Pseudocanthotermes spiniger*, namely, termicin, an antifungal peptide that contains cysteine and is constitutively present in hemocyte granules and the salivary glands of that termite and spinigerin, a cysteine-free peptide that is active against bacteria and fungi.

Termicin has little structural similarity with defensins from insects and its antimicrobial activity is more similar to sapecin B from *Sarcophaga peregrina*[22]; its activities are similar to that of defensins from plants and to that of the defensins drosomycin and heliomicin from the fly *D. melanogaster* and the lepidopteran *H. virescens*, respectively. Native termicin acts against fungal hyphae of *Aspergilus fumigatus* and against *C. albicans* and *C. neoformans* by reducing the hyphae length and increasing their branching. Recombinant termicin is active against *Saccharomyces cerevisiae*[4] [Table 2].

**Table 2:** Antimicrobial activity of defensins from invertebrates

*Name*	*Origin*	*Action*	*Similarity*	*Reference*
Cecropin	*Hyalophora ecropia, Aedes aegypti*	Antibacterial, Antifungal		23
Drosomycin	*Drosophila melanogaster*	Antibacterial		24
Thanatin	*Podisus maculiventris*	Antibacterial, Antifungal		25
Proline-rich peptides	Hymenoptera, Diptera, Lepdoptera, Hemiptera	Gram-negative bacteria, Antifungal		21
Attacins, Sarcotoxins II,	Diptera, Lepdoptera	Gram-positive bacteria		21
Diptericin				
Gloverin	*Hyalophora cecropia* (Lepdoptera)	Antimicrobial		26
Moricin	*Bobyx mori* (Lepdoptera)	Antimicrobial		27
Heliomicin	*Heliothis virescens*	Antifungal	Defensins from insects	4
Termicin	*Pseudocanthotermes, spiniger,* (termite)	Gram-positive bacteria Antifungal	Defensins from plants, Drosomycin, Heliomicin, Sapecin B	4
Spinigerin	*Pseudocanthotermes, spiniger,* (termite)	Antibacterial, Antifungal		4
Sapecin B	*Sarcophaga peregrina*	Antibacterial	Termicin	22

## Defensins from Plants

Defensins from plants have the ability to induce the flux of ions through the plasmatic membrane of fungal hyphae. However, in contrast to defensins from mammals and insects, they do not form pores in artificial membranes nor do they change the electric property of the lipoid artificial bilayer. Defensins from plants can inhibit a wide range of fungi.[28]

Two groups, A and B, with 25% of analogy, constitute the defensins from plants. Group A is subdivided into four subfamilies, A_1_, A_2_, A_3_, and A_4_, with a minimum of 50% of similarity. Group A_2_, formerly designated as non-morphogenic, which includes Dm AMP1 (from *Dahlia merckii*), reduces hyphal length without affecting the fungi morphology.[29] Groups A_3_ and A_4_, which include Rs-AFP2 (from *Raphanus sativus)*[30] and Hs-AFP1 (from *Heuchera sanguinea*), induce termination of hyphae and the branching of susceptible fungi, respectively, both being designated as morphogenic.[28]

Thevissen and collaborators[28] reported a dose-dependent effect of plant defensins on *Neurospora crassa*. With respect to *S. cerevisiae*, two types of membrane permeabilization were observed: (i) permeabilization of cation-sensitive membrane, induced by defensins from plants in cells suspended in water or in culture medium; (ii) permeabilization of cation-resistant membrane, only observed with Dm-AMP1, which inhibits *S. cerevisiae* [Table 3].

**Table 3:** Antimicrobial activity of defensins from plants

*Name*	*Origin*	*Action*	*Similarity*	*Reference*
Hs -AFP1 (A4)	*Heuchera sanguinea*	Antifungal	A2, A3 and A4 with 50%	28
Dm AMP1 (A2)	*Dahlia merckii*	Antifungal (*S cerevisiae*)	A2, A3 and A4 with 50%	29
Rs-AFP2 (A3)	*Raphanus sativus*	Antifungal	A2, A3 and A4 with 50%	30

## Bacteriocins

Bacteriocins are antibacterial, heterogeneous peptides characterized by their ability to inhibit almost all bacteria; they constitute a highly specific bactericide group, similar to the antibiotics. They have been shown to be generated by some samples of bacteria and only act on microorganisms intimately related to the producers. Pelczar and collaborators[1] reported that colicin, obtained from *E. coli*, was the first bacteriocin studied; it was followed by others namely: acrocin (*Enterobacter aerogenes*), pesticin (*Yersinia pestis*), marcescin (*Serratia marcescens*), piocin (*P. aeruginosa*). The other recently identified bacteriocins (with their respective producers) reported by Whitford and collaborators[31] are: lactococcin A (*Lactobacillus lactis*), thermophilin A (*Streptococcus thermophilicus*), and bovicin (*Streptococcus bovis*).

Inhibitory substances similar to the bacteriocin BLIS are found in bacteria from the rumen, the main producer being strains of *Streptococcus bovis* such as LRC 0253, LRC 0255, and LRC 0476; these strains produced BLIS that inhibited 16, 15, and 24 other *Streptococcus* strains from the rumen, respectively. All of them have similar characteristics to class II bacteriocin from Gram-positive bacteria.

Kemperman and collaborators[32] described the four classes of bacteriocins as follows: class I (including antibiotics) contains posttranslationally modified residues similar to lanthionine, β-methyl lanthionine, and dehydrated residues; class II does not have these modifications and are generally linear peptides; class III includes large-sized thermolabile bacteriocins; class IV is formed by complex molecules of proteins and other chemical compounds.

Bacteriocins of subclass IIc (class II bacteriocins or pediocin) are formed by circular molecules. Bacteriocins with such structure are: microcin J25 produced by *E. coli*, gassericin A produced by *Lactobacillus gasseri,* and AS-48/Bac21 produced by *Enterococcus faecalis.*[32]

Lüders and collaborators[33] investigated the combinations of antimicrobial eukaryotic peptides (AMPs) like pediocin PA-1,[34,35] sakacin P, and curvacin A[36] and pleurocidin[37] with prokaryotic AMPs. These latter are not being used commercially but have, however, been tested in medicine, mostly against Gram-negative and Gram-positive bacteria and sometimes also against other kinds of microorganisms. Prokaryotic AMPs have a narrow inhibitory spectrum and high antimicrobial potency. In that study[33] the authors detected a strong synergy with pleurocidin, which acts by permeabilizing the target-membrane to bacteriocins. Further studies have been carried out to find out the ideal AMPs concentrations necessary (along with pleurocidin) for inhibiting the growth of *E. coli*.

Two new broad-spectrum bacteriocins have been discovered:[37] enterocin L50A and L50B produced by *Enterococcus faecium* L50. The authors suggest that these two new bacteriocins, along with staphylococcal cytolysins, together constitute a new family of peptide toxins that are unrelated to class II bacteriocins because (i) they are excreted without a leader sequence or signal peptide; (ii) apparently, the structural genes of enterocins are not cotranscribed with a gene encoding an immunity protein; (iii) the organization of the enterocin L50 gene differs from class II bacteriocins gene clusters because, in addition to the bacteriocins gene(s), it includes genes encoding proteins involved in cell protection, processing, secretion, and regulation; (iv) the enterocins inhibit growth of a wide range of Gram-positive bacteria; and (v) they are synthesized as inactive precursors.[31]

These enterocins do not have hemolytic activity, which was reported to exist in broad-spectrum bacteriocins against *L. monocytogenes*, *S. aureus*, *B. cereus*, *C. perfringens,* and *C. botulinum.*[36,37]

The search for new bacteriocins is being intensively conducted. Two peptides with antimicrobial activity were isolated from a pathogenic and toxicgenic *Clostridium* found in spoiled food.[38] The authors described two new peptides: closticin 574 (from *Clostridium tyrobutyrium* ADRIAT 932), a class II bacteriocin similar to enterocin P, divergicin A, and listeriocin, which are generally secreted when the microorganism is in water; the other peptide isolated was circularin A (from *Clostridium beijerinckii* ATCC 25752), with a broad antibacterial activity, similarly to microcin J25, gassericin A, and AS-48. A new class (class V) was suggested for these bacteriocins due to their specific characteristics.

Another bacteriocin, the enterolysin A (Ent A), was obtained by purification from *Enterococcus faecalis* LMG culture 2333.[39] It inhibits the growth of bacteria of the genera enterococcus, pediococcus, lactococcus, and *Lactobacillus*, thus presenting the antimicrobial characteristic of class III bacteriocins.[40] [Table 4].

**Table 4:** Antimicrobial activity of bacteriocins

*Name*	*Origin*	*Action*	*Similarity*	*Reference*
Colicin	*E coli*	Antimicrobial		41
Acrocin	*Enterobacter aerogenes*	Antimicrobial		1
Pesticin	*Yersinia pestis*	Antimicrobial		42
Marcescin	*Serratia marcascens*	Antimicrobial		43
Pediocin	*Pseudomonas aeruginosa*	Antimicrobial		44
Lactococcin A	*Lactobacillus lactis*	Antimicrobial		45
Thermophilin A	*Streptococcus thermophilicus*	Antimicrobial		46
Bovicin	*Streptococcus bovis*	Antimicrobial		31
Microcin J25	*E coli*	Antimicrobial		47
Gassericin A	*Lactobacillus gasseri*	Antimicrobial		48
AS 48/Bac 21	*Enterococcus faecalis*	Antimicrobial		49
Pediocin PA-1, Sakacin P, Curvacin A, Pleurocidin	Eukariotes, Prokariotes	Antimicrobial		33
AMPs + Pleurocidin		*E.coli*		50
Enterocin	*E faecium L50*	*L. monocytogenes, S. Aureus, Bacillus cereus, Clostridium perfringens, Clostridium botulinum*		38, 39
Closticin 574	*Clostridium tyrobutyrum ADRIAT 932*	Antibacterial		32
Circularin A	*Clostridium beijerinckii*	Antibacterial	Microcin J25, Gassericin A, AS- 48	32
Enterolysin A	*Enterococcus faecalis LMG cultura 233*	*Enterococcus, Pediococcus, Lactococcus, Lactobacillus*		40

## Conclusion

Proteins and peptides with antimicrobial activity are found in different living organisms (humans, animals, and plants). It has been shown that several organisms produce peptides when induced by chemical or natural agents; these peptides act against microorganisms or even against predators; for example, termites produce a salivary substance that serves to protect their eggs.

Promising antimicrobial substances are being discovered and tested to evaluate their usefulness and safety for treating infectious diseases in humans, animals, and plants.

We beleive that researches carried out on the biological, therapeutic, and pharmacological aspects of defensins will aid our understanding of these proteins and their actions on organisms. We hope this review provides useful information for health professionals dealing with microbial control and resistance of organisms to drugs, especially antibiotics.[50]
